# Impact of the COVID-19 pandemic on lung cancer treatment and research

**DOI:** 10.1016/j.gendis.2023.01.028

**Published:** 2023-03-24

**Authors:** Shreya Kailas Lovlekar, Yihua Wang

**Affiliations:** Biological Sciences, Faculty of Environmental and Life Sciences, University of Southampton, Southampton SO17 1BJ, UK

Despite recent advances in immunotherapy and targeted therapy, the mortality rate seen in lung cancer (LC) is the highest of all cancer forms. The recent topical coronavirus disease 19 (COVID-19) and its subsequent pandemic have resulted in over 505 million confirmed cases and approximately 6.2 million fatalities as of April 2022,^1^ with a staggering 30%–50% mortality rate seen in LC patients with COVID-19.^2^ Cancer patients, in particular, are highly vulnerable to COVID-19 infection due to immunosuppression, from both the tumor and its treatments. Here we report the impact of the pandemic to be largely negative on LC treatment and research.

## Impacts on lung cancer treatments

**Figure 1:**
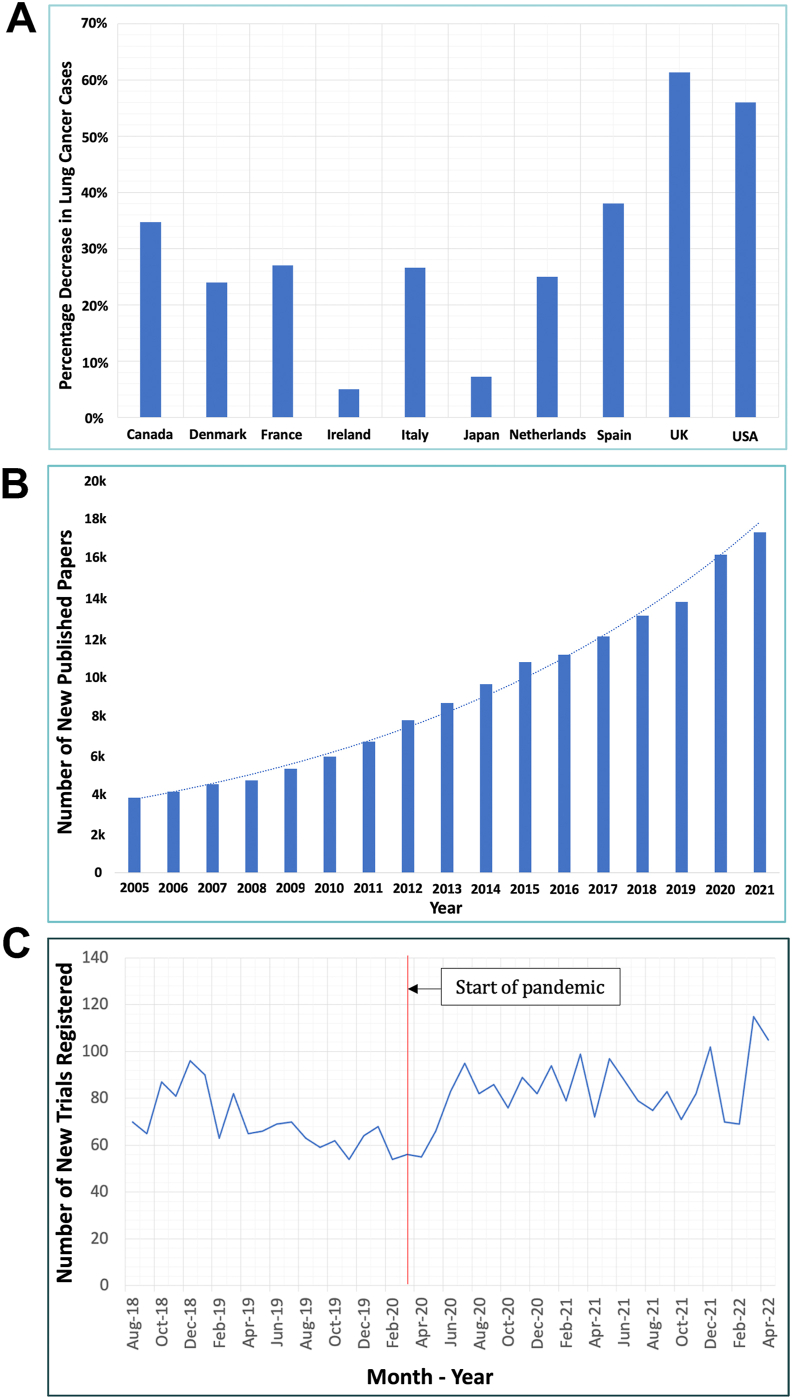
Impact of COVID-19 on lung cancer treatment and research. **(A)** Visualization of the percentage decrease in the number of lung cancer cases in various countries during the approximate March–May period in 2020 when compared with the baseline number of cases from previous years during the same March–May period. **(B)** Visualization of the trend in the number of new papers published annually on lung cancer on PubMed from 2005 to 2021. The red arrow indicates the start of the pandemic in 2020. Data were obtained from PubMed using the search criteria “lung cancer”. **(C)** Visualization of the trend in the number of new lung cancer trials registered on clinicaltrials.gov each month from August 2019 to April 2022. The red line denotes the official start of the COVID-19 pandemic in March 2020.

As seen in Figure 1A, many countries saw a significant decline in observed LC cases during the first peak pandemic period. Cancer care was also notably affected – a global study by Jazieh et al^3^ compiled data from 356 centers across 54 countries to gauge this effect. Although this study focuses on cancer care in general, rather than providing a breakdown of individual cancer types, it provides insight into reasons why care may have been affected. Most centers (around 64%) remained open over the pandemic but at suboptimal capacity and reported various reasons for this such as precautionary measures, staff shortages, and overburden to the system.^3^ Nearly half the centers also reported a shortage of personal protective equipment (PPE). Around 9% of centers were either fully or partially closed and the rest remained open at full capacity. Most centers in the study kept most services either partially or fully available, but of those that fully stopped, surgery appears to be the most disrupted across centers and systemic therapy the least. This is likely due to the increased risk of infection and immunosuppression that accompany surgery. However, the extent to which services were partially available was not quantified and thus the magnitude of disruption cannot be fully concluded. Jazieh and colleagues^3^ also found the distribution of disruption was relatively equal in countries across all levels of income; however, only 9 of the 356 centers were at the low-income level, so the conclusion that low-income countries were not affected disproportionately cannot be applied to all low-income countries.

## Impacts on trials and research

A search for the term “lung cancer” was conducted using the PubMed database, and the number of papers published each year was plotted on a graph (Fig. 1B). This search revealed no significant change to the overall trend in publications on lung cancer following the start of the pandemic in 2020; however, this does not necessarily mean that research was unaffected whatsoever. Over the pandemic, the sheer volume of COVID-19-related literature rapidly accumulated, with over 132,000 publications on PubMed in 2021 alone – 8 times as many as those on lung cancer. Whilst this is understandable given the topical nature of COVID-19, it may have resulted in some publications of lesser reliability and quality when compared with pre-pandemic levels due to the relaxation or even the absence of vigorous peer-reviewing processes.^4^ The same could extend to publications on lung cancer, meaning further assessment of literature published over the pandemic must be carried out to ensure their quality and trustworthiness.

Another search on PubMed for papers including both “lung cancer” and “COVID-19” yielded a total of 698 results, from 2020 to the present. This number is significantly lesser than the number of results for “COVID-19”, which may indicate the neglection of lung cancer in terms of its relevance to COVID-19 as a respiratory illness, despite the severity of lung cancer and COVID-19 comorbidity.

In terms of trials, a search on the clinical trials database (clinicaltrials.gov) for “lung cancer” studies revealed no negative impacts on the number of new trials being set up over the pandemic when compared with pre-pandemic figures (Fig. 1C), although there was a global decline of 14% in clinical trial participation over the peak of the pandemic.^5^ However, trials can take years to complete and thus the completion and relative success of these trials cannot yet be determined at present.

## Conclusions

Overall, the COVID-19 pandemic has had a distinctly negative impact on the lung cancer community, with increased morbidity and mortality, as well as a reduction in trial participation. Many countries saw a sharp decline in LC cases and there was also much public anxiety about attending in-person appointments, supplemented by conflicting messages from the government and lung cancer organizations that contributed to this decrease. The effectiveness of telehealth, which was outside the scope of this review, is another possible aspect that may have improved or worsened the delivery of care.

Further global collaborative studies need to be conducted in the near future to determine how different countries have been impacted based on income, the magnitude of research, the availability of specialist centers, and the relative number of COVID-19 cases. The focus should also be on if racial/ethnic disparities in the lung cancer community were further amplified due to the pandemic. Additionally, more funding and resources should be given to lung cancer trials and research, particularly for developing more early detection methods, as this is proven to significantly reduce mortality rates.

The volume of research paper publications may not have been outwardly affected, but the reliability and quality of a small number remain in question due to more lenient peer-reviewing processes. Furthermore, the data reviewed were from a singular database, *i.e*., PubMed, and thus evaluation of data from multiple databases should be considered in the future when determining the impacts on published literature. There may also be a delayed effect on clinical trials, and subsequent approval of therapies, due to the lockdowns and reduction in trial participation. Whilst the pandemic restrictions now appear to be lifting around the world and most people are immunized, there are still cases of COVID-19 present which could impact the unvaccinated and immunocompromised, especially those with lung cancer. A slight relaxation of lung screening criteria may aid in the detection of missed cases but does pose a risk of increasing false-positive diagnoses. It could also expose those with a slightly lower risk of lung cancer to ionizing radiation, meaning the risks outweigh the possible benefits. Regardless, the effects of missed cases will surely be seen in the next 5–10 years, with an increased number of deaths; however, if novel therapies currently in development are approved in time, this may change.

## Conflict of interests

For the purpose of open access, the authors have applied a CC-BY public copyright license to any Author Accepted Manuscript version arising from this submission. The authors declare that they have no relevant conflict of interests.

## Funding

YW was supported by the 10.13039/501100000265Medical Research Council (UK) (No. MR/S025480/1) and an 10.13039/501100000691Academy of Medical Sciences/the 10.13039/100010269Wellcome Trust Springboard Award (UK) (No. SBF002\1038).
